# Field evaluation of the efficacy of common anthelmintics used in the control of gastrointestinal nematodes of sheep in Dabat district, Northwest Ethiopia

**DOI:** 10.1186/s13620-017-0097-6

**Published:** 2017-06-07

**Authors:** Zewdu Seyoum, Yitayew Demessie, Basazinew Bogale, Achenef Melaku

**Affiliations:** College of Veterinary Medicine and Animal Sciences, University of Gondar, Gondar, Amhara Ethiopia

**Keywords:** Anthelmintic efficacy, Dabat district, FECRT, Gastrointestinal nematode, Sheep

## Abstract

**Background:**

Gastrointestinal nematode (GIN) infections are the main impediments that restrict the welfare and productivity of small ruminant in the world. Effective management of GINs in grazing sheep relies heavily on the use of highly efficacious anthelmintic drugs. However, anthelmintic resistance is becoming a significant concern in the world, and this phenomenon severely threatens the potential utilisation of this control strategy. Therefore, this study was conducted 1) to evaluate the efficacy of commonly used anthelmintic on GINs in naturally infected sheep and 2) to assess the farmers’ perception on anthelmintics utilisation practices in Dabat district, Northwest Ethiopia.

**Methods:**

One hundred twenty nematode infected sheep were used in this study. Sheep were selected based on the egg count (≥150 eggs per gram of faeces). The animals were allocated randomly into four groups (30 animals per group). Group-I, II and III were treated with Albendazole, Tetramisole, and Ivermectin, respectively. The 4th group was left untreated (as control). Faecal samples were collected on day 0 (before treatment), on day 3, 7, 10 and 14 (post-treatment). The modified McMaster technique was used for quantifying the eggs. Faecal egg count reduction test (FECRT) was applied to determine the efficacy of anthelmintic at day 14 (post-treatment). In addition, a questionnaire survey was conducted on 100 randomly selected sheep owners.

**Results:**

All anthelmintics tested revealed significant (*P* < 0.05) reduction in nematode egg excretion in the sheep post-treatment. Faecal egg count reduction (FECR) levels for Albendazole, Tetramisole, and Ivermectin were 97.2, 98.9 and 97.7%, respectively. Post-treatment egg counts and percentage reduction of egg counts were not significantly different (*P* > 0.05) among the treatment groups. The nematode genera identified before treatment were *Haemonchus*, *Trichostrongylus*, *Cooperia*, *Trichuris*, *Teladorsagia*, *Bunostomum,* and *Strongyloides. Haemonchus* and *Trichostrongylus* were detected after treatment with Albendazole and Ivermectin. The questionnaire survey revealed that Albendazole was the most commonly (90%) used anthelmintic to treat nematodes in sheep, followed by Tetramisole (36%) and Tetraclozan (Tetramisole-Oxyclozanide combination) (20%). Respondents expressed that anthelmintic selection was made based on veterinarian prescription (84%), colour (27%), efficacy (4%), price affordability (1%) and availability (1%).

**Conclusion:**

This study demonstrated that the tested anthelmintics had an acceptable level of efficacy against GINs of sheep.

## Background

Gastrointestinal nematode (GIN) infections remain as the most important impediments affecting small ruminants worldwide. Nematodes are responsible for both direct and indirect economic losses through decreased productivity, costs of control measures and deaths [1–3]. The control of parasitic nematodes in small ruminants relies largely on the use of anthelmintics [4–7]. However, anthelmintic resistance in GINs of small ruminants have been increasingly reported worldwide [5, 6, 8–10], and is severely threatening the potential utilisation of this control strategy [3, 7, 11, 12].

In Ethiopia, nematode infection in sheep is mainly controlled by the use of anthelmintics [2, 13]. Anthelmintic efficacy can be influenced by many factors; of which, under-dosing, frequent and indiscriminate use of drugs are the important factors that reduce the efficacy of anthelmintics [11, 14, 15]. The use of anthelmintic with substandard quality compounds [2, 16] and irrational use of anthelmintics [11, 14] can also influence the anthelmintic efficacy. Moreover, misuse and smuggling of anthelmintic drugs in many forms, such as illegal trading in open markets and irrational administration are widespread in Ethiopia due to an absence of a rational policy for anthelmintic use [14, 17, 18]. Furthermore, methods that can preserve and prolong the efficacy of anthelmintics and prevent the emergence of anthelmintic resistance have never been practised in many parts of the country [2, 19, 20].

Broad spectrum anthelmintics such as Albendazole (Benzimidazoles group), Tetramisole (Imidazothiazoles group), and Ivermectin (Macrocyclic lactones group) have been used for the last four decades to treat and control nematode infections in sheep in Ethiopia. These drugs are imported and distributed by different agencies in the country [18, 21]. Despite the use of Albendazole, Tetramisole, and Ivermectin for a considerable period of time, there is a paucity of information on the efficacy of these anthelmintics in the Northwest part of Ethiopia. Thus, the objectives of the present study were to assess the perception of sheep owners about their anthelmintic utilisation and to evaluate the efficacy of the most commonly used anthelmintics against GINs in naturally infected sheep at field level in Dabat district, Northwest Ethiopia.

## Methods

### Study area

The study was carried out from November 2015 to April 2016 in Dabat district, North Gondar Zone of Amhara Regional State, Northwest part of Ethiopia. The district has highland, midland, and lowland agroecologies. It lies at a latitude of 12.4°N and longitude of 27.25°E with an altitude of 1500–3200 m above sea level. The annual average minimum and maximum temperatures of the district are 18 °C and 35 °C, respectively. The annual rainfall of the area ranges from 800 to1400 mm. The area has two main seasons: the wet (rainy) season, which extends from June-September when the area receives the majority of its rainfall and a dry season from October to May; rainfall is erratic and low. The majority of the communities in the district are involved in the animal production and the area has an estimated 81,000 sheep population owned by 26,775 farmers (average flock size = 3) [22]. In the district, three specific sites, namely Weken, Chilla and Dabat Zuria were purposively selected based on accessibility and the presence of a sufficient sheep population with a history of frequent use of anthelmintics for the control of internal parasites.

### Questionnaire survey

A semi-structured questionnaire (with open and closed-ended questions) was prepared and 100 sheep owners were interviewed in order to get information on the anthelmintics utilisation and perceived efficacy. The sample size of the respondents was determined using the formula (*n* = 0.25/SE^2^) proposed by Arsham [23] at the standard error (SE) of 0.05 with 95% confidence interval. Animal health workers and community leaders were also involved in the selection of sheep owners. Field assistants (enumerators) with knowledge of animal health were hired in order to support and carry out the interviews. Before the interview, the objective of the research was explained to each respondent and the full consent of the participant was obtained. Each interview was completed within 30 to 35 min. The questionnaire focused mainly on information on the commonly used anthelmintics, the frequency of use, criteria for selection, main source, and rotation of anthelmintics, observations on the responses of treatment (efficacy) and educational background of each participant.

### Experimental animals and design

The study animals were locally bred, kept under an extensive husbandry system and maintained on communal grazing land with access to the same watering points. At night sheep from each farm were kept in pens at their owners’ respective houses. For screening purposes, a total of 228 female sheep (age: 1–2 years old) were selected from 40 different flocks. Each sheep for inclusion in the study was tagged in the left/right ear bearing a unique identification number. Criteria for inclusion included the following: sheep that had not received any anthelmintic in the previous 12 weeks, a minimum flock size of 20 sheep, farmers’ willingness to participate and history of anthelmintics usage and a faecal egg count (FEC) ≥ 150 eggs per gram of faeces. Faecal samples were collected per rectum from each sheep and FEC determined using the McMaster technique as described by Taylor et al. [24].

A total of 120 naturally nematode infected female sheep were identified and included in the study. The identified sheep were randomly allocated to four groups of 30 sheep. Group-I animals were treated with Albendazole, Group-II with Tetramisole, Group-III with Ivermectin and Group-IV was left untreated (control). Animals were treated according to their body weight with the dose recommended by the manufacturer. The research team members were assigned per site to conduct the sample collection. At each sampling site, field assistant was also assigned to support the team members. Faecal samples were then collected per rectum from each animal on the same day in all the study sites before (at Day 0) and after treatment (at 3, 7, 10 and 14 days) according to the recommendation of Coles et al. [25]. Samples were placed in individually sealed containers, labelled with the sheep tag unique number and placed in the cool ice box and transported without delay to the Parasitology Laboratory, CVMAS, UoG for a faecal examination. Upon arrival at the laboratory, the samples were kept at 4 °C in the refrigerator until processing (i.e. FEC and coproculture).

### Faecal egg count

The McMaster counting technique was carried out for each faecal sample in order to determine the number of eggs per gram of faeces (EPG) [26]. Briefly, 3 g of the faecal pellet was mixed in 42 ml of saturated NaCl solution with a sensitivity of 50 EPG of faeces [27].

### Faecal egg count reduction test (FECRT)

The FECRT and the 95% confidence intervals for the reduction estimates were calculated according to the methods described in the World Association for the Advancement of Veterinary Parasitology (WAAVP) recommendations for the detection of anthelmintic efficacy or resistance in ruminants [24, 25, 27].

### Coproculture and Larval identification

Faecal samples contained nematode eggs from each group of animals were pooled, finely disrupted using a mortar and pestle, and cultured in Wide-Mouthed Jar for larval identification [28]. A small amount of water was added to moisten the faecal sample and it was left at room temperature for 14 days mixing periodically to avoid fungal growth. Then, the larvae were collected following the procedures described in Bayou [28] and MAFF [29]. After collection of the larvae, the third stage larvae were mounted on slides, killing using Lugol’s iodine and identified under a microscope to the genus level in each group (before and after treatment) based on morphological characteristics as described by VanWyk et al. [30].

### Data analysis

All data were entered into Excel spreadsheets. The data were analysed using Statistical Package for Social Sciences (SPSS) V-20 statistical software [31]. Descriptive statistics (percentages) were used to measure the results describing the respondents’ responses to the questionnaire. Results are presented as percentages and the absolute numbers on which these percentages are in parentheses from a questionnaire survey. The reduction in FEC post-treatment was calculated using 100 (1- Xt/Xc) where the Xt arithmetic means of post-treatment egg count on the 14th day and Xc arithmetic mean of the control group at 14th day [25]. The log transformation of the values of EPG [using log (x + 1)] was performed to minimise and stabilise the variance. A 95% confidence interval was calculated as follows:

$$ \begin{array}{l}\mathrm{Upper}\ \mathrm{confidence}\ \mathrm{limit} = 100\ \left[1\hbox{-}\ \mathrm{Xt}/\ \mathrm{Xcexp}\ \left(\hbox{-} 2.04{8}^2\right)\right]\\ {}\mathrm{Lower}\ \mathrm{confidence}\ \mathrm{limit} = 100\ \left[1\hbox{-}\ \mathrm{Xt}/\ \mathrm{Xcexp}\ \left(+2.04{8}^2\right)\right]\\ {}\mathrm{Where}\ {\mathrm{Y}}^2\mathrm{denotes}\ \mathrm{the}\ \mathrm{variance}\ \mathrm{of}\ \mathrm{the}\ \mathrm{reduction}\end{array} $$

Reduction in egg counts of less than 95% and with lower 95% confidence limit less than 90% was considered as indicative of resistance against the drug [25].

## Results

### Questionnaire survey

**Fig. 1:**
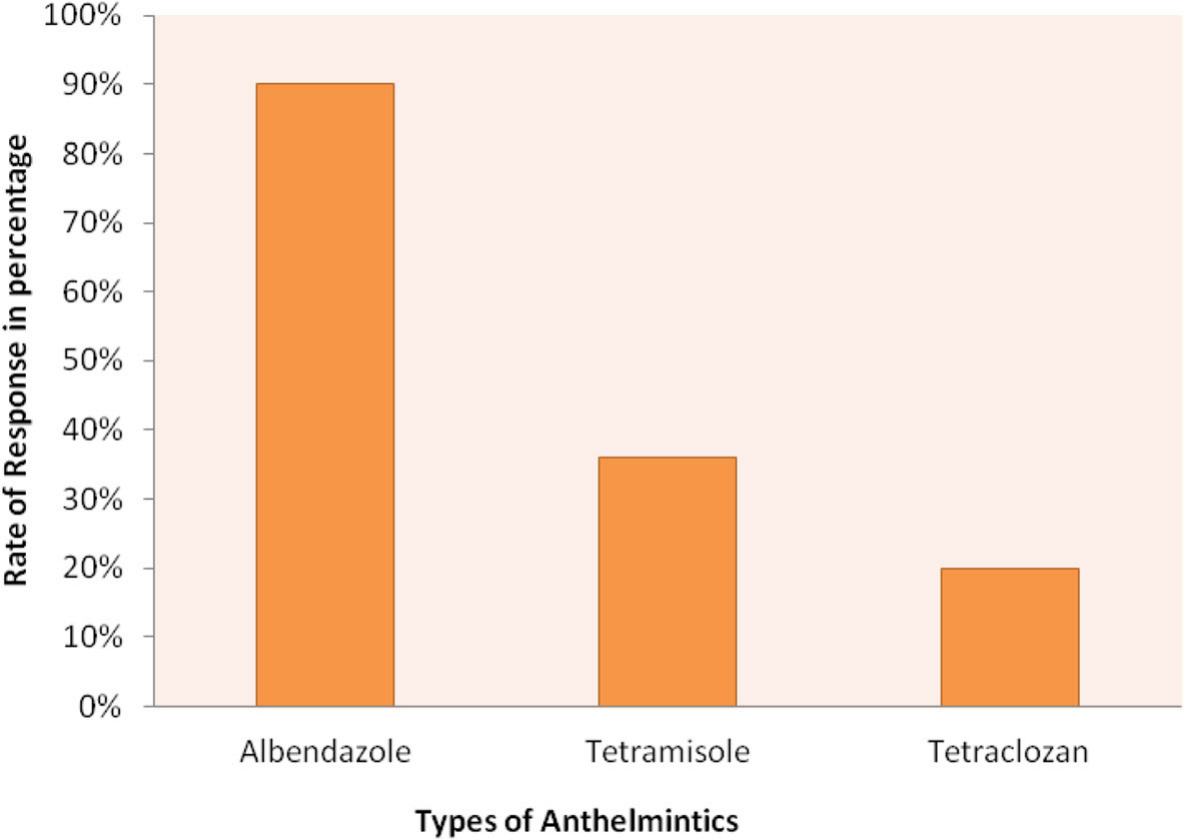
Overall anthelmintic preferred by respondents

All respondents (100) indicated that they practised anthelmintic treatment to control internal parasites of the sheep. Most of the respondents reported that Albendazole (Benzimidazoles group) was the more commonly used anthelmintic in sheep followed by Tetramisole (Imidazothiazoles group) and Tetraclozan (Tetramisole-Oxyclozanide combination) (Fig. 1). Respondents recognised these groups as ‘green, white and pink with groove ’ respectively. According to the results, 42% [32] of respondents were illiterate, 37% [33] were grade 1–4, 13% [13] were grade 5–8 and 8% [8] were above grade 9. Regarding the source of anthelmintics, 70% [70] of the respondents obtained from the nearby government-owned veterinary clinic, 23% [23] from an open market/shop and 7% [7] from private veterinary pharmacies. Farmers have selected anthelmintics based on prescription by veterinarians (84%), colour (27%), efficacy (4%), price affordability (1%) and availability (1%).

The farmer’s reasons for treating their animals with anthelmintics was varied; approximately a third of respondents [34] treated because of respiratory signs (coughing and nasal discharge), 27% [24] were related to general disease symptoms (emaciation, rough hair coat, weakness and loss of condition). Moreover, 27% [24] of respondents stated that they have used for a simple deworming purpose without clinical signs and 14% [14] utilised against digestive disturbance signs (diarrhoea and reduced appetite). Regarding treatment frequency, 49% [35] of farmers have treated their sheep twice a year, 39% [36] three times a year and 12% [12] once a year.

### Dose determination methods, knowledge of anthelmintics rotation and administration

A large proportion of respondents (78% [78]) indicated that they determined the dose rate for their sheep based on the prescribing advice of the animal health professionals while the remainder of participants (22% [22]) determined the dose rate based on a visual appraisal of the weight of the animal. The majority of owners (94% [94]) responded that they had no knowledge or experience of rotating their anthelmintics. Most of the respondents (98% [98]) administered anthelmintics to their sheep directly themselves, whereas the remainder had veterinary support. Following anthelmintic treatment; 96% [96] and 93% [93] of participant declared an improvement in the clinical signs and body condition of their sheep respectively.

### Faecal egg counts (FEC) and FECR

**Fig. 2:**
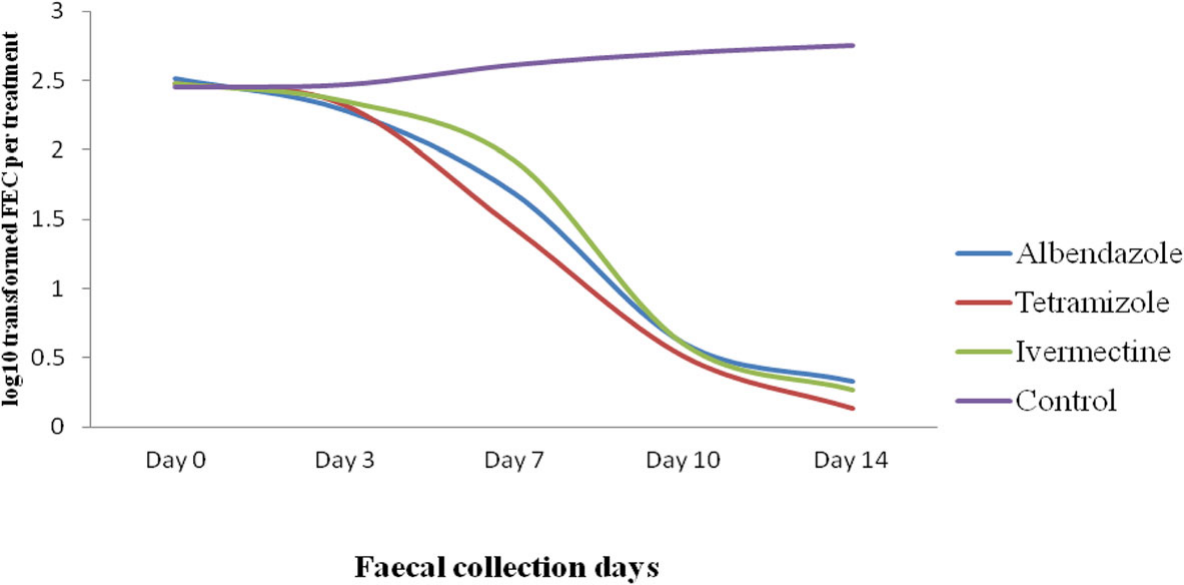
FEC reduction percentage with three anthelmintics on GINs of sheep in sampled days

The anthelmintics that were chosen for testing were based on the frequency of utilisation in the area. The details of the drugs used in the tests are summarised in Table 1. FEC results from the 3 study sites pre and post treatment with the three anthelmintics are shown in Tables 2 and 3. The FEC did not differ between sites (*P* > 0.05). The mean FEC between groups (treated and control non-treated) was not significantly different before treatment (day zero). On day 14, post-treatment egg counts and percentage reduction of FEC or FEC were not significantly different (*P* > 0.05) among the three anthelmintic treated groups. All anthelmintics exerted a significant (*P* < 0.05*)* reduction effect on nematode egg counts post-treatment (Table 3). As indicated in Fig. 2, all anthelmintics exerted their effects on egg count output starting from day 3.

**Table 1:** Description of the anthelmintic drugs used in the FECRT for field efficacy trial

Family Name	Generic name	Trade name	Manufacturer	Dose (mg/kg BW)	Route	Manufactured date	Expired date
Benzimidazoles	Albendazole	Ashialben 300 mg	Ashish Life Science Private Limited, India	7.5	Per os	09/01/2014	08/01/2018
Imidazothiazoles	Tetramisole	Doxam 600 mg	Chengdu Quiankun, Veterinary, Pharmaceuticals, Co. Ltd. China	15	Per os	01/09/2014	22/08/2017
Macrocyclic lactones	Ivermectin	Ivermectin injection 1%	Hebei New Century Pharmaceutical Co., Ltd., China	0.2	Subcutaneous	16/01/2015	15/01/2018

**Table 2:** Faecal egg counts pre and post treatment in sheep studied from 3 sites in Dabat district of NW Ethiopia

Study site	Treatment								
	Albendazole			Tetramisole			Ivermectin		
	Day0	Day14	%FECR	Day0	Day14	%FECR	Day0	Day14	%FECR
Weken	320 ± 103.3	20 ± 42.2	96.6%	360 ± 126.5	0.0 ± 0.0	100%	320 ± 103.4	20 ± 42.2	96.6%
Dabat Zuria	360 ± 126.5	20 ± 42.2	96.6%	300 ± 133.3	10 ± 31.6	98.3%	330 ± 125.2	10 ± 31.6	98.3%
Chilla	360 ± 135.0	10 ± 31.6	98.3%	310 ± 99.4	10 ± 31.6	98.3%	300 ± 81.7	10 ± 31.6	98.3%

**Table 3:** Mean faecal egg counts of nematodes in sheep before and after treatment

Anthelmintic	Mean FEC ± SEM					EPG reduction	95% CI	
	Day 0	Day 3	Day 7	Day 10	Day 14		Lower	Upper
Albendazole	346.7 ± 119.6	216.7 ± 102	110 ± 71.2	33.3 ± 54.7	16.7 ± 37.9	97.2	93.3	98.8
Tetramisole	323.3 ± 119.4	236.7 ± 109.8	103.3 ± 89	27 ± 51.9	6.7 ± 25.4	98.9	95.3	99.7
Ivermectin	316.7 ± 102	243.3 ± 93.5	133.3 ± 71.1	30.0 ± 46.6	13.3 ± 34.6	97.7	94.0	99.1
Control	300.0 ± 105.1	313.3 ± 113.7	436.7 ± 147.5	513.3 ± 119.6	586.7 ± 165.6	NA	NA	NA

*FEC* Faecal egg count, *SEM* standard error of the mean, *NA* not applicable

### GIN Identification

The faecal culture of eggs to third stage larvae was undertaken parallel to faecal egg counting to differentiate the type of nematodes before and after treatment in each anthelmintic treatment and the control group. Overall, the parasite genera identified (but not quantified) before treatment irrespective of a group were *Haemonchus*, *Trichostrongylus*, *Cooperia*, *Trichuris*, *Teladorsagia*, *Bunostomum,* and *Strongyloides. Haemonchus* species were observed after treatment with Albendazole and Ivermectin while a small number of *Trichostrongylus* species were detected after treatment with Ivermectin (Table 4).

**Table 4:** Identified nematode genera in cultures of faecal samples before and after treatment

Anthelmintics		Survived parasite
Before treatment		*Haemonchus, Cooperia, Trichuris, Trichostrongylus, Teladorsagia, Bunostomum* and *Strongyloides*
After treatment	Albendazole	*Haemonchus and Trichostrongylus species*
	Tetramisole	None
	Ivermectin	*Haemonchus species*

## Discussion

In the present study, the result indicated that Albendazole was the most widely-used anthelmintic followed by Tetramisole. This finding is in agreement with reports from southern Ethiopia by Kumsa and Nurfeta [2] and from north-west Ethiopia by Melaku et al. [34]. In this study, the majority of respondents (84%) indicated that they have been administering anthelmintics to their sheep by professional prescription, which agrees with previous work by Terefe et al. [14] from Oromia Region, Ethiopia. Forty-nine percent of respondents stated that they have dewormed their sheep twice a year; while 39% of owners treated three times a year. This is in line with the finding of Melaku et al. [34] in Northwest Ethiopia. Most the respondents indicated that their animals displayed improvement on both clinical signs and body condition after treatment. This supports the report of Datiko et al. [37] who stated that 81% of the respondents indicated that their animals have shown improvement in both clinical signs and body condition after treatment.

The results on anthelmintic efficacy were interpreted according to the WAAVP recommendations [24, 25]. All three anthelmintics; Albendazole, Tetramisole, and Ivermectin were effective against gastrointestinal nematodes. This finding is supported by previous studies on gastrointestinal nematodes of sheep kept under extensive husbandry by smallholder farmers in Ethiopia [13, 14, 38] and in sheep under controlled conditions [2]. This result is also in agreement with the findings of Kumsa and Wossene [19] who reported that Albendazole and Tetramisole were highly efficacious against *Haemonchus contortus* in experimentally infected lambs.

While Albendazole has been the most frequently used anthelmintic in Ethiopia [2, 20, 21, 37], previous experimental and field studies on the efficacy of this drug in Ethiopia have shown variable results. Satisfactory efficacy levels (≥95%) of Albendazole on nematodes have reported by various authors [2, 17, 18, 20, 21, 39] and thus agree with the current study findings. In contrast, the results of other studies indicate inefficacy of Albendazole treatment, which might be due to resistance development on Albendazole [33, 40, 41]. Moreover, this might be ascribed to the factors like quality of generic repacked or reformulated products and management, quality of drugs [16, 36], which might be responsible for these varied reports. Resistance to a benzimidazole group of anthelmintics has now been widely reported and in particular where sheep production is more intensive [9, 10]. The differences observed between studies might be attributed to the production system and environment. In an extensive production system, the level of drug selection on worm population is much lower since a majority of parasites being in refugia, and lower frequency of treatments [33].

In the present study, Ivermectin was also found to be effective against gastrointestinal nematodes on 14th-day post-treatment with a 97.72% percentage reduction in egg counts. This is in line with the studies conducted on nematodes of sheep maintained under the extensive system by smallholder farmers in Ethiopia [2, 17, 18, 39]. Similarly, the finding of this study also supported by previous reports from different parts of the world [16, 32, 42, 43]. However, the result obtained in this study contrasts previous studies conducted by Kumsa and Abebe [33] and Getachew et al. [41] from Ethiopia with 63–84.44% FECR. The report of this study is also in contrast to the reports of Traversa et al. [44] from Italy with 88% FECR and Pena-Espinoza et al. [45] from Denmark with 71% FECR in lambs at field condition. The observation of anthelmintic resistance in nematodes in these studies is most probably due to the combination of selection factors like high frequency of treatments, simultaneous and indiscriminate use of the same drugs and under dosage treatment practices by owners; all of which are reported to favour and enhance anthelmintic resistance development [15, 16, 36, 43, 45].

Similar to the observation in this study, Tetramisole was reported as a favourite for use against helminth parasites in Ethiopia by Eguale et al. [46]. Previous studies have shown variable effects of Tetramisole against parasitic nematodes [17, 39, 47]. In this study, Tetramisole was found to have a relatively higher efficacy, which is in agreement with the reports of studies in Ethiopia by Kumsa and Nurfeta [2], Getachew et al. [41] and Sibhatu et al. [48]. In contrast, Asmare et al. [17] from Southern Ethiopia reported 97.5% FECR with 85% lower limit of 95% confidence interval and Melaku et al. [34] from Northwest Ethiopia with 84.87% FECR and 73.95% lower limit of 95% confidence interval for Tetramisole. This variation in the efficacy of anthelmintics at different localities may be due to the occurrence of resistant nematode strains, dosing errors and perhaps low-quality products [21].

In the present study, the nematode genera identified before treatment were similar to that found by other Ethiopian based studies and studies conducted worldwide [14, 15, 33, 35, 40, 49]. While *Haemonchus and Trichostrongylus* species were the only nematodes remained post-treatment. This finding supports previous reports [2, 15, 21]. This finding is also consistent with that of Hamdullah et al., [50] from Pakistan. This might be due to the greater ecological and biological plasticity of these parasites [16, 21].

## Conclusion

This study demonstrated that the tested anthelmintics had an acceptable level of efficacy against GINs of sheep. While the present study indicated all three anthelmintics were highly efficacious; but is in contrast to other Ethiopian studies. This is a very good indication to avoid the fear of anthelmintic resistance. Nationwide studies with standardised protocols are necessary to determine the status of the efficacy of the commonly used anthelmintics in various agroecology, management systems and species of animals.
